# Mismatch Repair Deficiency Profiling and Its Impact on Management and Prognosis in Endometrial Cancer Patients: A Comprehensive Update

**DOI:** 10.7759/cureus.99364

**Published:** 2025-12-16

**Authors:** Emmanouela-Aliki Almperi, Chrysoula Margioula-Siarkou, Aristarchos Almperis, Georgia Margioula-Siarkou, Stefanos Flindris, Alexandros I Daponte, Theodora Papamitsou, Konstantinos Dinas, Stamatios Petousis

**Affiliations:** 1 2nd Department of Obstetrics and Gynaecology, Gynaecologic Oncology Unit, Ippokrateio General Hospital of Thessaloniki, Aristotle University of Thessaloniki, Thessaloniki, GRC; 2 2nd Department of Obstetrics and Gynaecology, Gynaecologic Oncology Unit, Aristotle University, School of Medicine, Thessaloniki, GRC; 3 Department of Obstetrics and Gynaecology, University of Thessaly, Larissa, GRC; 4 Laboratory of Histology-Embryology, School of Medicine, Faculty of Health Sciences, Aristotle University of Thessaloniki, Thessaloniki, GRC

**Keywords:** cancer immunotherapy, endometrial cancer (ec), mmr deficient, molecular profile, platinum based chemotherapy

## Abstract

With an increasing incidence, endometrial cancer (EC) is the most prevalent gynecologic cancer in developed countries. Although histological classification has been used traditionally, its usage in forecasting clinical behavior was less effective. A molecular classification of EC has been introduced by the Cancer Genome Atlas (TCGA), which has separated it into four subgroups: p53-abnormal (p53abn), mismatch repair deficiency (MMRd), polymerase epsilon (POLE)-mutated (POLEmut), and no specific molecular profile (NSMP). The prognostic value of this molecular subtyping is superior. This narrative review analyzes the clinicopathological features of MMRd tumors, highlighting their intermediate prognosis relative to other molecular subtypes, while also noting associations with high-grade, lymphovascular space invasion, and lymph node metastasis. For this purpose, relevant studies were retrieved from PubMed, Scopus, and Web of Science up to 2025, focusing on clinicopathological correlations and treatment outcomes. Furthermore, we analyze the important therapeutic implications of MMR status. About 25%-30% of cases are classified as MMRd EC and are associated with an intermediate prognosis. The increased mutational burden in MMRd tumors enhances their susceptibility to immune checkpoint inhibitors such as pembrolizumab and dostarlimab, which have demonstrated considerable efficacy and altered the treatment approach for patients with advanced or recurrent MMRd EC. This review highlights the significance of MMR testing in risk stratification, prognosis, and planning of personalized treatment, ultimately improving patient outcomes.

## Introduction and background

Endometrial cancer (EC) stands as the most prevalent malignancy of the female reproductive system in developed countries, with incidence rising 1%-2% annually. Although it is typically diagnosed in postmenopausal women, individuals with certain predisposing factors such as obesity, family history of EC, early menarche, late menopause, advanced age, and infertility are also at high risk for developing. With no screening test available, diagnosis relies on high clinical suspicion and is confirmed by endometrial biopsy [1]. Due to the fact that the majority of cases are diagnosed at early stage, the five-year overall survival rate (OS) is estimated at 95% for patients with local disease and 69% for those with regional disease, whereas the rate decreases to 17% in approximately 8%-10% of patients presented with advanced or recurrent disease, mainly attributed to the lack of effective systemic treatment.

EC staging relies on a combination of imaging and histological evaluation to indicate the tumor’s size, depth of myometrial invasion, nodal involvement, and presence of distant metastases. Traditionally, EC has been categorized into subtypes: type I or endometrioid (EEC) and type II or non-endometrioid (NEEC) subtype, with the former presented typically as low-grade and often linked to estrogen excess, obesity, hyperplasia and a more favorable prognosis [2]. Αlthough widely used, this dualistic system has limited prognostic value, as histology, grade, and stage alone do not fully capture clinical behavior. The publication of The Cancer Genome Atlas (TCGA) in 2013, detailing the genomic landscape of endometrial carcinoma led to a revolution of EC characterization with the adoption of a molecular classification. More specifically, using immunohistochemistry (p53, MSH6, PMS2) and polymerase epsilon (POLE) mutation testing, ECs can be divided into four biologically distinct groups: POLE-mutated (POLEmut), mismatch repair deficiency (MMRd), p53-abnormal (p53abn), and no specific molecular profile (NSMP) and finally categorized into four prognostic risk groups as low-, intermediate-, intermediate/high, and high-risk, with only 5% of endometrial carcinomas, termed “multiple classifiers”, exhibiting overlapping molecular features. POLEmut patients are associated with the most favorable prognosis, whereas p53abn with the poorest one. Mismatch repair deficiency (MMRd) is caused by the biallelic inactivation of an *MMR* gene, which can be attributed either to an inherited or an acquired pathway. MMRd is characterized by loss of nuclear immunostaining for at least one of the four MMR proteins that are routinely examined: MSH2, MSH6, MLH1, and PMS2 and results in microsatellite instability (MSI), a condition of mutation accumulation and elevated neoantigen production which enhances tumor immunogenicity and sensitivity to immune checkpoint inhibitors [3].

Recognizing the expanding role of molecular profiling in risk stratification and therapeutic decision-making in endometrial cancer, main objective of the present narrative review is to critically summarize current evidence regarding correlation of MMR deficiency with histopathological parameters, prognostic implications, as well as the impact on survival outcomes of patients with endometrial cancer, to optimize individualized treatment approaches. For this purpose, relevant studies were retrieved from PubMed, Scopus, and Web of Science up to 2025, focusing on clinicopathological correlations and treatment outcomes.

## Review

Implementation of molecular classification in clinical practice

Risk stratification based on molecular classification has recently been integrated into the European (European Society of Gynaecological Oncology (ESGO), European Society for Radiotherapy and Oncology (ESTRO), and European Society of Pathology (ESP)) guidelines for management of EC, and molecular characteristics now play a critical role in estimating recurrence risk and, consequently, overall survival outcomes. The updated clinical approach increasingly incorporates detection of MMR deficiency or microsatellite instability (MSI) in preoperative biopsies, using immunohistochemistry (IHC) to evaluate loss of MMR proteins [4]. While IHC is widely favored due to its accuracy, cost-effectiveness, and suitability for small biopsy samples and demonstrates high concordance with polymerase chain reaction (PCR) tests (about 96%), it may occasionally miss MSI cases detectable by PCR or next-generation sequencing (NGS) [5]. Despite these limitations, current guidelines endorse IHC as the primary diagnostic method. Although the majority of EC cases are sporadic, approximately 5%-10% are hereditary, often associated with Lynch syndrome (LS), which is characterized by a germline mutation in the mismatch repair genes. In nearly half of women with LS, EC tends to arise as the first malignancy, at a younger age, which underscores the importance of heightened vigilance in detecting LS, particularly in women under 50 years of age presenting with EC. The molecular screening methods include MMR-IHC, MSI test, and *MLH1* promoter methylation test and gene sequencing. Because *MLH1* loss or high microsatellite instability (MSI-H) can reflect either sporadic *MLH1* hypermethylation or germline MMR pathogenic variants (Lynch syndrome), universal *MLH1* promoter methylation test is recommended to exclude sporadic EC [6]. Consequently, literature strongly supports universal tumor screening coupled with genetic counseling for all endometrial cancer patients to identify Lynch syndrome, which accounts for 3%-5% of EC cases to ensure optimal therapeutic results.

Histopathological and molecular correlates of MMR deficiency

Τhe MMR-deficient group, initially identified by TCGA as the "hypermutated group," exhibits a high mutational load, although lower than that of the ultramutated/POLE-mutated group. Τhe reported prevalence ranges from 24.5% (95% CI: 21.0%-28.1%) in a meta-analysis of 7,459 patients to 27.0% in a cohort of 163 patients with endometrioid EC [7]. Accounting for 28% of EC cases, all hypermutated ECs demonstrated high microsatellite instability, indicative of rapid genomic mutation accumulation. Since microsatellite instability is commonly due to a deficiency in the MMR system, immunohistochemistry for MMR proteins has been utilized as a surrogate test to identify this group. Τhe assessment involves four MMR proteins: MLH1, MSH2, MSH6, and PMS2. Loss of MLH1 or MSH2 leads to subsequent loss of PMS2 or MSH6, respectively. Conversely, loss of MSH6 or PMS2 expression can occur independently. Evaluating MSH6 and PMS2 alone has been proposed as having equivalent accuracy to the full MMR panel in identifying MMR-deficient cases. However, assessing MMR immunohistochemical expression can be challenging and may be influenced by fixation issues. A positive internal control in endometrial stroma and lymphocytes is crucial, with normal expression characterized by tumor cell nuclei positivity stronger than stromal positivity [8].

Ιndeed, the MMR-deficient group primarily comprises endometrioid carcinoma (85.8%), with a notable prevalence of high-grade cases (47.4%) [8]. This deficiency is significantly more common in high-grade compared to low-grade endometrioid carcinomas (39.7% vs. 24.7%) and is particularly prevalent in undifferentiated/dedifferentiated carcinoma (44%) and mixed ΕCs with an endometrioid component (16%-66%). Conversely, its occurrence is lower in clear-cell carcinoma (9.8%) and carcinosarcoma (7.3%) [9]. ΜMRd ΕCs with serous morphology and/or immunophenotype are often considered endometrioid EC [9,10]. Additionally, MMRd ECs frequently exhibit prominent lymphocytic infiltration and significant morphological heterogeneity [9].

Lymphovascular Space Invasion (LVSI)

Lymphovascular space invasion (LVSI) is significantly more frequent in MMRd tumors compared to mismatch repair pro (MMRp) tumors. In a cohort of 312 EC patients, LVSI was observed in 27.2% of MMRd cases versus 16.9% of MMRp cases (p = 0.02) [11]. Similarly, in a study of 163 endometrioid EC patients, LVSI was present in 54.8% of MMRd tumors compared to 25.0% of MMRp tumors (p = 0.001) [7]. Multivariate analysis confirmed MMRd as an independent risk factor for LVSI, with a relative risk (RR) of 3.44 (95% CI: 1.45-8.16; p = 0.005) [7].

Myometrial Invasion

Τhe depth of myometrial invasion is a critical prognostic factor in EC. Αmong MMRd tumors, myometrial invasion of less than 50% was observed in 51.8% (95% CI: 38.5%-64.9%), while invasion greater than 50% was reported in 42.3% (95% CI: 28.6%-56.8%) [9]. In another study, 65.1% of MMRd tumors had less than 50% myometrial invasion, compared to 78.2% in MMRp tumors (p = 0.092) [7].

Lymph Node Metastasis

Lymph node metastasis is more common in MMRd tumors. In a cohort of 163 endometrioid EC patients, lymph node metastasis was observed in 22.7% of MMRd cases compared to 7.6% of MMRp cases (p = 0.008) [7]. Para-aortic lymph node involvement was also higher in MMRd tumors (18.2% vs. 4.2%, p = 0.031) [7]. Multivariate analysis identified deep myometrial invasion as the only independent risk factor for lymph node metastasis (RR: 9.15, 95% CI: 2.74-30.57; p < 0.001), while MMRd showed a marginal association (RR: 2.85, 95% CI: 0.95-8.57; p = 0.062) [7].

The overall prognosis of ΜMRd ECs falls into an intermediate category. While MΜRd prognostically surpasses p53 abnormalities, it is overshadowed by POLE mutations [3]. Compared to POLE-mutant ECs, MMRd ECs appear to be more influenced by clinicopathological variables, although not to the extent of NSMP ECs. MMRd ECs are stratified into different risk groups based on International Federation of Obstetrics and Gynecology (FIGO) grade, histotype, depth of myometrial invasion, and LVSI according to ESGO-ESTRO-ESP guidelines. However, the prognostic significance of these factors in MMRd ECs is unclear. For instance, while deep myometrial invasion and LVSI significantly worsen the prognosis of MMRd ECs, tumor grade appears to lack independent prognostic value. Histotype may also lack prognostic value in MMRd ECs. Instead, MMR deficiency consistently associates with an intermediate prognosis across different histotypes, resulting in worsened outcomes in early-stage, low-grade ECs and improved outcomes in non-endometrioid ECs [12]. These clinicopathological correlations can be supported by the results of the most recent meta-analysis that included both retrospective and prospective data [7]. However, undifferentiated/dedifferentiated carcinoma, which constitutes about two-thirds of such cases, often exhibits the loss of crucial proteins of the switch-sucrose non-fermentable (SWI/SNF) complex, leading to exceedingly poor prognosis even in the presence of an MMR-deficient signature. Maybe traditional histopathological parameters lose independent prognostic value in MMRd endometrial cancer because the hypermutated genomic landscape and immunogenic tumor microenvironment fundamentally override morphological features as the primary determinants of clinical behavior and therapeutic response. A summary of MMRd clinicopathological features can be found in Table 1.

**Table 1:** MMR-Deficient EC: Clinicopathologic Features MMR: mismatch repair, EC: endometrial cancer, MMRd: mismatch repair deficiency, MMRp: mismatch repair pro, RR: relative risk, LVSI: lymphovascular space invasion, LN: lymph node, MI: myometrial invasion.

Feature	MMRd Findings	Comparison to MMRp	Statistical Significance
Histology	• 85.8% endometrioid • 47.4% high-grade • 44% undifferentiated/dedifferentiated • Lower in clear cell (9.8%) and carcinosarcoma (7.3%)	• More common in high-grade vs low-grade endometrioid • Serous-like MMRd often reclassified as endometrioid	• 39.7% vs 24.7% in high-grade vs low-grade (p < 0.05) • Prominent lymphocytic infiltration and heterogeneity characteristic
LVSI	• 27.2%-54.8%	• 16.9%-25.0% in MMRp	• p = 0.001-0.02 • RR: 3.44 (95% CI: 1.45-8.16) • MMRd = independent risk factor for LVSI
Myometrial invasion	• <50%: 51.8%-65.1% • ≥50%: 42.3%-34.9%	• <50%: 78.2% in MMRp • ≥50%: 21.8% in MMRp	• p = 0.092 (not significant) • Trend toward deeper invasion in MMRd
Lymph node metastasis	• Overall: 22.7% • Para-aortic: 18.2%	• Overall: 7.6% in MMRp • Para-aortic: 4.2% in MMRp	• p = 0.008 (overall LN) • p = 0.031 (para-aortic) • RR: 2.85 (95% CI: 0.95-8.57, p = 0.062) • Deep MI = strongest predictor (RR: 9.15)

Prognostic implications of MMR deficiency

The four *MMR* genes exhibit distinct prognostic patterns in endometrial cancer. MLH1 promoter hypermethylation paradoxically confers the most favorable prognosis, typically presenting in older patients with lower-grade tumors. Among Lynch syndrome cases, MSH6 mutations are most common and carry intermediate prognosis with later-onset disease, while MSH2 mutations demonstrate more aggressive behavior with earlier presentation, non-endometrioid carcinoma, and higher extrauterine spread. PMS2 mutations (~5%) show the mildest phenotype with best outcomes. However, whether these differences reflect true biological distinctions or confounding by age, surveillance bias, and tumor grade remains controversial, as Lynch-associated cases often benefit from earlier detection through screening programs.

Overall Survival (OS) and Progression-Free Survival (PFS)

The mean overall survival (OS) and progression-free survival (PFS) for MMRd EC patients vary across studies. In a cohort of 312 patients, the median follow-up was 54.5 months (range: 0-120.5 months), and no significant difference in OS was observed between MMRd and MMRp groups [11]. In another study, MMRd tumors with MLH1/PMS2 loss were associated with reduced PFS (p = 0.008), while other MMRd subgroups showed similar PFS to MMRp tumors [7]. Cox regression analysis identified lymph node metastasis as the only independent risk factor for reduced PFS (p = 0.004), with MLH1/PMS2 loss showing marginal significance (p = 0.054) [7].

Recurrence

Recurrence rates are lower in MMRd tumors compared to MMRp tumors. In a study of 312 EC patients, recurrence occurred in 6.9% of MMRd cases versus 14.8% of MMRp cases (p = 0.02). However, recurrence-free survival (RFS) did not differ significantly between MMRd and MMRp groups (HR: 0.93, 95% CI: 0.59-1.47). MMR-deficient ECs have demonstrated higher susceptibility to radiotherapy compared to MMR-proficient ECs. Due to their high mutational load and immune infiltrate, ΜMR-deficient ΕCs are potential candidates for immunotherapy [7].

Therapeutic impact of MMR status

Chemotherapy and Hormonal Therapy

Platinum-based chemotherapy remains the standard first-line systemic therapy beyond endocrine therapy for advanced or recurrent EC. Therapeutic options after this are associated with poor outcomes with response rates of 20% or less. Currently, conventional chemotherapy (ChT) remains the primary treatment approach for recurrent or metastatic endometrial cancer. Another option involves hormone therapy as a first-line treatment. Its efficacy has primarily been observed in low-grade endometrioid tumors and is dependent on the status of estrogen receptor (ER) and progesterone receptor (PgR). However, its association with treatment response quality is not as well-established as in breast cancer. Resistance to chemotherapy has been documented in MMRd tumors, which could potentially account for the poorer prognosis observed in the advanced stage compared to MMRp tumors. This can occur because deficient mismatch repair (dMMR) system fails to recognize drug-induced DNA damage, preventing apoptosis activation, while the hypermutated state enables selection of chemoresistant clones with mutations in drug metabolism and transport pathways [13]. The available evidence regarding the sensitivity of endometrial cancer (EC) to chemotherapy based on MSI or MMR status is lacking or insufficient. According to the Retrospective Analysis of the Correlation of MSI-h/dMMR Status and Response to Therapy for Endometrial Cancer (RAME) study, an interesting finding was that, in patients with advanced/recurrent disease, first-line platinum-based chemotherapy was connected with longer PFS and OS in the microsatellite instability-low (MSI-l)/proficient mismatch repair (pMMR) population compared to MSI-h/dMMR patients [14].

Immunotherapy and Clinical Trials 

Tumors exhibiting deficient mismatch repair (dMMR) system or high microsatellite instability (MSI-H) demonstrate elevated tumor mutational burden (TMB) and extensive regions of genomic instability. This heightened neoantigen load, triggering a robust immune response and consequently such tumors are predictive of response to immune-based therapies. ICIs are particularly effective in dMMR/MSI-H tumors because of their high mutational burden, which increases the production of tumor-mutated antigens, such as programmed death-ligand 1 (PD-L1). ICI target the PD‐1/PD‐L1 pathway, a T‐cell inhibitory signaling pathway, enhancing the efficacy of anticancer immunotherapy. In May 2017, pembrolizumab received accelerated FDA approval for refractory MSI-H/dMMR solid tumors, including endometrial cancer, marking the first tissue-agnostic approval based on a common biomarker rather than tumor origin. The approval was supported by compelling clinical data demonstrating a 36% objective response rate in heavily pretreated MSI-H patients, with durable responses ranging from four to 17+ months [15]. Supporting the approval were findings from KEYNOTE-158, which demonstrated an objective response rate (ORR) of 48% (95% confidence interval (CI), 37%-60%) and a progression-free survival (PFS) of 13.1 months (95% CI: 4.3-34.4 mοnths) in pretreated patients with MMR-D/MSI-H tumors [16]. Interim analysis of the GARNET trial evaluating dostarlimab, an anti-PD1, on 104 patients with endometrial cancer who had disease progression after at least one line of chemotherapy, showed an ORR of 43.5% (95% CI: 34%-53.4%) with a 12-month duration of response in 93.3% of patients. This led to FDA approval for use in endometrial cancer patients with recurrent and/or advanced disease after experiencing progression with platinum-based chemotherapy [17]. Similar response rates were observed in one additional study within the subset of patients with MMR-D/MSI-H endometrial cancer patients who had received prior therapy and examined treatment with durvalumab in that cohort [18].

Across four trials evaluating immune checkpoint inhibitors for MMRd endometrial cancer, significant improvements in progression-free survival (PFS) and overall survival (OS) were observed compared to placebo. In the AtTEnd trial, atezolizumab plus chemotherapy achieved a PFS HR of 0.36 (95% CI: 0.23-0.57, p = 0.0005) and an OS HR of 0.82 (95% CI: 0.63-1.07, p = 0.048), demonstrating a 64% reduction in progression risk and a modest survival benefit [19]. The RUBY trial with dostarlimab showed similar efficacy, with a PFS HR of 0.42 (95% CI: 0.22-0.80) and an OS HR of 0.59 (95% CI: 0.42-0.83), highlighting durable responses [20]. In the GY018 trial, pembrolizumab combined with chemotherapy yielded a PFS HR of 0.30 (95% CI: 0.19-0.48, p < 0.001), representing a 70% reduction in progression risk, although OS data remains immature [21]. The DUO-E trial evaluated durvalumab with or without olaparib, achieving a PFS HR of 0.55 (95% CI: 0.42-0.70) and an OS HR of 0.59 (95% CI: 0.42-0.83, p=0.003), indicating a 45% reduction in progression risk and improved survival [22]. Comparisons between MMRd and MMRp groups were not consistently reported, but where available, MMRd patients derived greater benefit. These results underscore the significant advantage of incorporating immunotherapy into first-line treatment for MMRd endometrial cancer [22].

Two other clinical trials, the DUO-E and ENGOT-En9/LEAP-001, provide substantial evidence regarding the efficacy of different therapeutic approaches in advanced or recurrent endometrial cancer according to MMR status. The DUO-E trial demonstrated statistically significant improvements in progression-free survival (PFS) with the addition of the anti-PD-L1 antibody durvalumab to first-line platinum-based chemotherapy (carboplatin/paclitaxel), followed by maintenance durvalumab with or without the poly (ADP-ribose) polymerase (PARP) inhibitor olaparib. Ιn MMRd patients, the PFS hazard ratios (HRs) favored the durvalumab arm (HR: 0.42, 95% CI: 0.22-0.80) and the durvalumab plus olaparib combination arm (HR: 0.41, 95% CI: 0.21-0.75). In MMRp patients, the addition of maintenance olaparib showed a clinically meaningful benefit when combined with durvalumab (HR: 0.57, 95% CI: 0.44-0.73) [22]. In contrast, the ENGOT-En9/LEAP-001 trial, which compared lenvatinib/pembrolizumab (LEN/PEMBRO) to standard chemotherapy in MMRp patients, did not achieve non-inferiority in overall survival (OS) (HR: 1.02, 95% CI: 0.83-1.26) or progression-free survival (HR: 0.91, 95% CI: 0.76-1.09). This could be attributed to their low tumor mutational burden and reduced immunogenicity, which limits ICI efficacy and necessitates multi-mechanistic approaches combining chemotherapy and PARP inhibitors to induce immunogenic cell death. However, LEN/PEMBRO exhibited durable responses in exploratory analyses, with a median duration of response (DOR) of 23.2 months compared to 10.6 months with chemotherapy. Both trials highlight stronger benefits in patients with MMRd tumors, confirming the utility of immunotherapy for this subset and signaling potential roles for PARP inhibitors as a maintenance therapy in MMRp subgroups. These findings underscore the value of molecular profiling in guiding first-line treatment for advanced EC [23]. A summary of the major clinical trials can be found in Table 2.

**Table 2:** Summary of Major Immunotherapy Trials MMR: mismatch repair, PFS: progression-free survival, OS: overall survival, ORR: objective response rate, a/rEC: advanced/recurrent endometrial cancer, chemo: chemotherapy, CI: confidence interval, D: durvalumab, D+O: durvalumab + olaparib, DOR: duration of response, EC: endometrial cancer, HR: hazard ratio, dMMR: deficient mismatch repair, MSI-H: high microsatellite instability, MMRp: mismatch repair pro.

Trial	Treatment Regimen	Population	MMR Status	PFS HR (95% CI)	OS HR (95% CI)	ORR (95% CI)	Key Findings
KEYNOTE-158 [16]	Pembrolizumab (mono)	Pretreated, tumor agnostic	dMMR/MSI-H	-	-	48% (37%-60%)	Median PFS: 13.1 months (4.3-34.4); FDA approval for MMR-D/MSI-H tumors
GARNET [17]	Dostarlimab (mono)	Pretreated EC, ≥1 prior line	dMMR/MSI-H	-	-	43.5% (34%-53.4%)	12-month DOR: 93.3%; FDA approval for recurrent/advanced EC post-platinum
AtTEnd [19]	Atezolizumab + chemo	First-line a/rEC	dMMR	0.36 (0.23-0.57)	0.82 (0.63-1.07)	-	64% reduction in progression risk; modest OS benefit
RUBY [20]	Dostarlimab + chemo	First-line a/rEC	dMMR	0.42 (0.22-0.80)	0.59 (0.42-0.83)	-	Durable responses; 41% reduction in death risk
GY018 (NRG-GY018) [21]	Pembrolizumab + chemo	First-line a/rEC	dMMR	0.30 (0.19-0.48)	Immature	-	70% reduction in progression risk; OS data pending
DUO-E [22]	Durvalumab ± olaparib + chemo	First-line a/rEC	dMMR	0.42 (0.22-0.80) [D] 0.41 (0.21-0.75) [D+O]	0.59 (0.42-0.83)	-	45% reduction in progression risk; no additional benefit with olaparib in dMMR
DUO-E [22]	Durvalumab + olaparib + chemo	First-line a/rEC	MMRp	0.57 (0.44-0.73) [D+O]	-	-	Clinically meaningful benefit with olaparib addition in MMRp
ENGOT-En9/LEAP-001 [23]	Lenvatinib + pembrolizumab	First-line a/rEC	MMRp	0.91 (0.76-1.09)	1.02 (0.83-1.26)	-	Did not meet non-inferiority; median DOR: 23.2 vs 10.6 months (chemo)

Moreover, a systematic review and meta-analysis examined the efficacy of immune checkpoint inhibitors (ICIs) and PARP inhibitors (PARPi) as first-line therapies for advanced or recurrent endometrial cancer (a/rEC) pointed out that ICI monotherapy remains the optimal approach with no added benefit of PARPi for MMRd patients, with significant gains in PFS and OS in mismatch repair-deficient (MMRd) tumors. Conversely, in MMRp tumors, particularly p53-abnormal/PD-L1-positive subgroups, combination therapies significantly delay recurrence and progression (HR: 0.47, 95% CI: 0.40-0.94). Patients treated with the combination experienced a 57.6% 12-month PFS (95% CI), which exceeded the 43.7% observed with ICI alone. However, p53-wildtype MMRp tumors did not demonstrate incremental benefits with PARPi addition, and outcomes favored ICI monotherapy, suggesting divergent reliance on DNA repair pathways for tumorigenesis in these subgroups [24].

Future Directions and Combination Strategies 

The evaluation of mismatch repair status serves three primary purposes. Firstly, it is utilized to screen patients for the possible diagnosis of Lynch syndrome. Secondly, it is employed to predict the response to PD-1 axis inhibition. Lastly, MMR status is used as a prognostic biomarker. Immune checkpoint inhibitors have received approval for use in advanced or recurrent endometrial cancer after prior systemic therapy. Nonetheless, ongoing studies are actively investigating their potential utilization in alternative clinical settings. For dMMR/MSI-H endometrial cancer, immunotherapy agents pembrolizumab and dostarlimab are currently FDA-approved for advanced/recurrent cases, and recent data from the GY-018 and RUBY trials suggest immunotherapy in combination with chemotherapy as front-line therapy is a promising option, as dostarlimab in combination with carboplatin-paclitaxel has demonstrated a significant substantial increase in progression-free survival at 24 months (61.4% in the dostarlimab group vs 15.7% in the placebo group, p < 0.001) [25]. Given the importance of these studies, a key next step is to mitigate the combined toxicities associated with immunotherapy and standard chemotherapy by adopting immunotherapy as an initial solo treatment and nοt only to thοse who have not responded to platinum-based therapy. To this direction, supporting data from two randomized clinical trials, GINECO-EN105b/ENGOT-en13/DOMENICA and KEYNOTE-C93/GOG-3064/ENGOT-en15, assess, respectively, dostarlimab and pembrolizumab in comparison to standard chemotherapy with the goal of de-escalating the first-line treatment for MMRd patients [26,27].

Beyond immune checkpoint inhibition, ongoing research is exploring PARP inhibitors, tumor vaccines, and adoptive T-cell therapies as potential options for MMRd endometrial cancer. The efficacy of combining ICIs with anti-angiogenic agents has been examined, specifically focusing on the pairing of lenvatinib and pembrolizumab for treating endometrial cancer, mainly concentrated οn the mismatch repair proficient subgroup though. In contrast, the KEYNOTE-775 trial revealed notable efficacy within the mismatch repair-deficient cohort [28]. These outcomes led to designate lenvatinib in conjunction with pembrolizumab as a breakthrough therapy for patients with recurrent or metastatic MMRp endometrial cancer following at least one prior systemic treatment [15]. In this context, according to the phase III ENGOT-En9/LEAP-001 trial (NCT03884101) pembrolizumab plus lenvatinib against the conventional regimen of paclitaxel and carboplatin in newly diagnosed stage III/IV or recurrent cases failed to meet the predefined thresholds for OS or PFS in the MMRp group, while the potential benefit in MMRd tumors remains uncertain, particularly concerning the justification of any increased toxicity. The comprehensive results are awaited for publication [23].

While immune checkpoint inhibitors have traditionally been utilized for advanced-stage disease and first recurrences, current research is now investigating their application in early-stage endometrial cancer, particularly for patients with MMRd status. Ongoing trials are evaluating the use of ICIs in adjuvant and neoadjuvant settings as well as their incorporation into fertility-sparing approaches for specific patient groups, with the aim of taking advantage of immunotherapy earlier in the treatment process and potentially lowering relapse rates and reducing reliance on chemoradiotherapy, while ensuring oncological safety [29]. Artificial intelligence and molecular imaging may also aid in early detection of recurrence and treatment response monitoring, a promising yet underexplored field. Future research should elucidate the optimal sequencing and duration of immunotherapy, mechanisms of acquired resistance, and the utility of biomarkers in directing therapeutic selection. Although the incorporation of molecular profiling, precision immunotherapeutic strategies, and translational research holds potential to transform endometrial cancer management, multidisciplinary collaboration and equitable access to diagnostic testing remain critical prerequisites for translating these scientific advances into improved patient outcomes worldwide.

## Conclusions

Τhe TCGA classification has provided a remarkable opportunity to enhance the risk stratification and management of EC. By grouping ΕCs based on molecular signatures, it may help reduce inconsistencies in assigning grades and histological subtypes, particularly for morphologically diverse and ambiguous ECs. Future studies should prioritize the identification of specific biomarkers that can forecast responses to immunotherapy and develop optimal combination strategies with other systemic therapies tailored to various molecular subtypes. Furthermore, the safety and effectiveness of immune checkpoint inhibitors (ICIs) in both adjuvant and neoadjuvant settings remains inadequately understood. Although initial findings from ongoing trials are encouraging, it is vital to persist in exploring innovative therapeutic combinations that address both primary tumors and metastatic disease, with the aim of decreasing recurrence rates and enhancing long-term patient outcomes. This review underscores the necessity of a multidisciplinary approach to integrate emerging molecular diagnostics with clinical practice, optimizing treatment decisions to ensure that patients receive the most effective personalized care possible. In any case, immunotherapy represents a significant advancement in the management of MMRd endometrial cancer, sometimes combined with other systemic therapies for different molecular subtypes, and ongoing research is crucial to fully realize its potential and establish new standards of care within this diverse disease landscape. However, further research is necessary to address remaining issues such as the sub-stratification of the NSMP group and the prognostic significance of clinicopathological variables in MMR-deficient ECs.
